# A genomics-informed, SNP association study reveals FBLN1 and FABP4 as contributing to resistance to fleece rot in Australian Merino sheep

**DOI:** 10.1186/1746-6148-6-27

**Published:** 2010-05-26

**Authors:** Wendy JM Smith, Yutao Li, Aaron Ingham, Eliza Collis, Sean M McWilliam, Tom J Dixon, Belinda J Norris, Suzanne I Mortimer, Robert J Moore, Antonio Reverter

**Affiliations:** 1 CSIRO Livestock Industries, 306 Carmody Rd, St Lucia, QLD 4067, Australia; 2 Industry and Investment NSW, Agricultural Research Centre, Trangie, NSW 2823, Australia; 3 SheepGENOMICS, Wool Sub-Program, 306 Carmody Rd, St Lucia, QLD 4067, Australia; 4 Australian Animal Health Laboratory, CSIRO Livestock Industries, Private Bag 24, Geelong, Vic. 3220, Australia

## Abstract

**Background:**

Fleece rot (FR) and body-strike of Merino sheep by the sheep blowfly *Lucilia cuprina *are major problems for the Australian wool industry, causing significant losses as a result of increased management costs coupled with reduced wool productivity and quality. In addition to direct effects on fleece quality, fleece rot is a major predisposing factor to blowfly strike on the body of sheep. In order to investigate the genetic drivers of resistance to fleece rot, we constructed a combined ovine-bovine cDNA microarray of almost 12,000 probes including 6,125 skin expressed sequence tags and 5,760 anonymous clones obtained from skin subtracted libraries derived from fleece rot resistant and susceptible animals. This microarray platform was used to profile the gene expression changes between skin samples of six resistant and six susceptible animals taken immediately before, during and after FR induction. Mixed-model equations were employed to normalize the data and 155 genes were found to be differentially expressed (DE). Ten DE genes were selected for validation using real-time PCR on independent skin samples. The genomic regions of a further 5 DE genes were surveyed to identify single nucleotide polymorphisms (SNP) that were genotyped across three populations for their associations with fleece rot resistance.

**Results:**

The majority of the DE genes originated from the fleece rot subtracted libraries and over-representing gene ontology terms included defense response to bacterium and epidermis development, indicating a role of these processes in modulating the sheep's response to fleece rot. We focused on genes that contribute to the physical barrier function of skin, including keratins, collagens, fibulin and lipid proteins, to identify SNPs that were associated to fleece rot scores.

**Conclusions:**

We identified FBLN1 (fibulin) and FABP4 (fatty acid binding protein 4) as key factors in sheep's resistance to fleece rot. Validation of these markers in other populations could lead to vital tests for marker assisted selection that will ultimately increase the natural fleece rot resistance of Merino sheep.

## Background

Fleece rot (FR) is a bacterial dermatitis of the sheep skin and fleece caused by an overgrowth of the natural skin microflora following prolonged exposure to moisture [1,2]. FR is characterised grossly by bands of matted and often discoloured fibres along the mid-line of the animal over the neck, wither, mid-back and rump regions. Its severity ranges from bacterial discoloration causing coloured bands (water stain), to extensive gummy exudates (wool rot) causing bands of matted fibres [3]. Severe FR can cause weakening of wool substantially lowering wool quality and value. In addition to direct effects on fleece quality, FR is the most important predisposing factor to blowfly body strike, a form of blowfly strike, in eastern Australia. Inflammation and ulceration of the skin occurs at the FR affected site attracting blowflies which deposit eggs and providing moisture for the eggs to hatch and soluble protein for the freshly hatched larvae to feed on which can lead to severe tissue damage and death in extreme cases. Body strike causes significant losses annually as a result of increased chemical and labour costs and reduced production.

Three host barriers have been identified that are involved in the development of resistance to blowfly strike: wool, skin and the immune system. Considerable examination of the influence of fleece production traits such as colour, wax content, fibre diameter and fleece weight on susceptibility to FR has been conducted [4,5]. Early morphological changes of the skin in response to wetting have been shown to include increased vascular permeability, infiltration of inflammatory cells and epidermal thickening [6,7]. The contribution of the skin barrier, bacterial communities and the sheep local skin inflammatory, innate and adaptive immune responses to FR susceptibility are less well studied and understood. Divergent serological responses against *Pseudomonas aeruginosa *[8] and immune-inflammatory responses [1,9,10] have been documented in FR and fly strike resistant (RES) and susceptible (SUS) animals. Ovine defence mechanisms associated with skin IgE+ cells were found to play an important role in resistance to fleece rot [9]. In contrast, T lymphocyte-dependent immunological effector mechanisms could not be found to affect the growth or survival of blowfly larvae [11]. Other than these studies reviewed by Norris et al (2008) [12], little is known about the host's response to FR and the underlying causes of susceptibility.

Divergent selection has demonstrated the potential for breeding animals with enhanced resistance to FR and fly strike (reviewed by Bishop and Morris (2007) [13]); however, conditions suitable for expression of resistance only occur sporadically in many environments. Therefore, knowledge of resistance genes and identification of molecular markers for resistance would provide the means for marker assisted selection or introgression of resistance across the Merino industry and lead to improved animal health and welfare, and reduced management costs and chemical residues.

The current experiment investigated the genetic drivers of resistance to FR using a three-step approach: First, a skin-focussed cDNA microarray was constructed and applied to RES and SUS animals to identify differentially expressed (DE) genes; Second, a selected group of DE genes was validated via qRT-PCR, and their coding regions surveyed to identify single nucleotide polymorphisms (SNP); Finally, these SNPs were genotyped across three populations with different FR characteristics to ascertain their association to FR resistance.

## Results

### Analysis of phenotypic data

**Figure 1:**
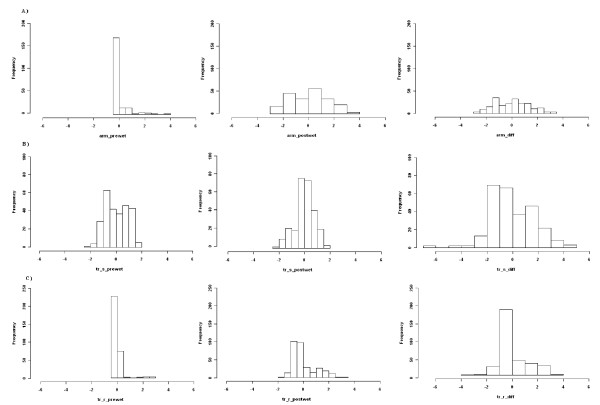
**The distribution of residual fleece rot scores determined in three flocks of sheep: A) Armidale Mapping Flock; B) Trangie Susceptible Line; C) Trangie Resistant Line**. For each flock the pre-wetting (left panels), post-wetting (middle panels) and difference between pre-wet and post-wet scores are presented (right panels). At each data collection time the sheep were scored using the conventional 0 to 5 scoring system. For each animal this score was then converted to a residual fleece rot score, by subtracting a correction factor that accounts for significant environmental and polygenetic effects. For example, if the original fleece rot score was 1 but the correction value for that animal was 3 the residual fleece rot score would be (1-3) or -2. Hence, the spread of residual scores does not fall within the original 0 to 5 range, but instead is scattered around zero.

Means (± standard error) for prewet FR scores were 0.17 ± 0.04, 1.75 ± 0.11, and 0.08 ± 0.03 for Armidale, Trangie RES and Trangie SUS flocks, respectively. The same set of values for the postwet FR scores were 2.24 ± 0.11, 3.09 ± 0.10, and 0.85 ± 0.07. The distributions of FR score residuals determined after adjusting for significant environment effects and polygenic components of inheritance are shown in Figure 1. Residual FR scores were determined at the pre-wetting and post-wetting sample collection times. A third score was generated by determining the difference between the pre- and the post-wetting scores and this identified animals that showed the greatest change in FR score over the time of the experiment.

Prior to the wetting trial, the Armidale flock had a low infection rate and produced FR scores that followed a Poisson distribution (Figure 1A). After exposing the sheep to conditions designed to induce FR, the scores were close to a normal distribution. A high incidence of FR was confirmed in the Trangie susceptible line (Figure 1B) at the pre-wetting sampling time. This was not the case for either the Armidale mapping flock (Figure 1A) or the Trangie RES line (Figure 1C). Following wetting, the increased FR susceptibility is evident from the distribution of both the post-wetting and the difference data that were skewed towards higher FR scores. Conversely, the Trangie RES line showed a distribution skewed towards lower FR scores, for both pre- and post-wetting trials (Figure 1C).

### Genes differentially expressed between RES and SUS sheep

**Figure 2:**
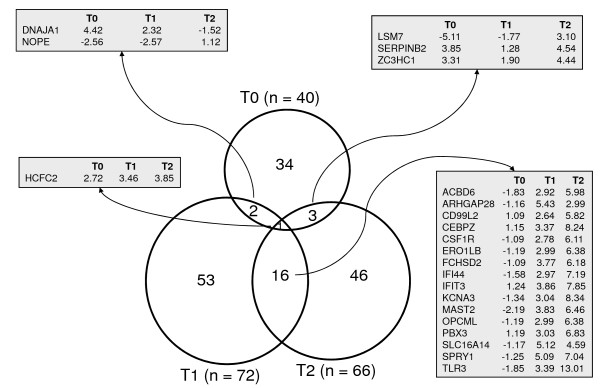
**Venn diagram relating the distribution of DE genes across three time points (T0, T1 and T2)**. The identity of genes found to be DE in more than one time point is given in the enclosed boxes along with their fold change expression between the RES and the SUS lines of sheep. For example, there are two genes (DNAJA1 and NOPE) that were DE at T0 and T1. DNAJA1 showed a significant 4.42 and 2.32-fold increase in RES sheep at T0 and T1, and a non-significant -1.52-fold decrease at T2. The size of the circles and amount of intersection relates to the number of DE in each area.

In total, 155 genes were identified as being DE between the RES and the SUS sheep at either one of the three sampling times: immediately before (T0), during (T1; two days into the trial) and after FR induction trial (T2; eleven days after the trial). The number of DE genes identified at each sampling time were 40, 72 and 66 at T0, T1, and T2, respectively (Figure 2). These numbers were above the expected number of significant calls (32) that could have been made by chance alone, from a total of 3,238 independent tests (individual genes) and at a 0.01 significance level. The corresponding false positive rate was estimated at 80%, 44% and 48% for T0, T1, and T2, respectively. However, due to genes interacting with each other, the number of independent tests must be less than the number of genes, meaning that these error rates specify the upper limit. Table S1 (Additional file 1) lists the set of 155 DE genes along with their normalized mean expression across the six experimental conditions (two genotypes, RES and SUS, at the three time points, T0, T1 and T2).

There was a smaller set of genes simultaneously DE at any two times. For example, DNAJA1 and NOPE were shared between T0 and T1, 16 DE genes were common to T1 and T2 lists, and three genes between T0 and T2. Only one gene, HCFC2, was DE at all time points, with expression levels consistently 2 to 4-fold higher in RES sheep.

**Figure 3:**
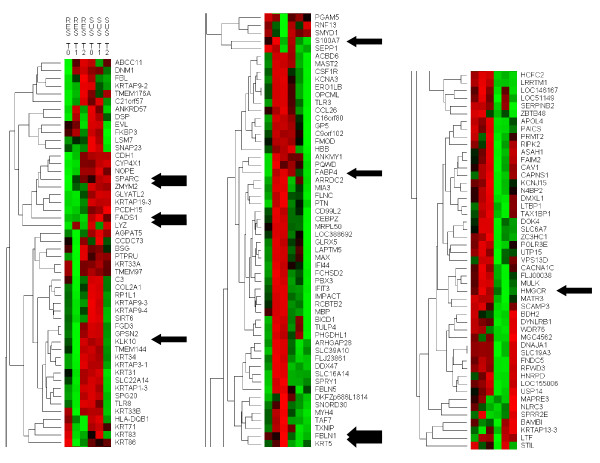
**Heat map of the hierarchical clustering of the 155 DE genes across the three time points (T0, T1 and T2) in each of the two lines (RES and SUS sheep)**. Green and red indicate low and high expression, respectively. Arrows indicate the location of genes that were further scrutinized by qRT-PCR validation and SNP association analyses.

The normalised expression of these genes determined in RES and SUS animals, at each of the three sampling times, is shown in the heat map of Figure 3. Hierarchical clustering was performed in order to identify groups of genes that behaved similarly across time points. Three large clusters were identified that were characterised as follows: 1) Genes that fall or are naturally lower in RES sheep but are higher or increase in SUS sheep (Figure 3, left panel); 2) Genes that rise in RES but fall in SUS sheep (Figure 3, middle panel); and 3) Genes that are naturally higher in RES but fall in SUS sheep (Figure 3, right panel). A number of smaller clusters existed within these three large super-clusters that may indicate functionally related sets of genes.

The analysis of gene ontology (GO) terms on the set of 155 DE genes resulted in the existence of four over-represented (P < 0.01) biological processes: 1) Visual behaviour (HMGCR - 3-hydroxy-3-methylglutaryl-coenzyme a reductase, DYNLRB1 - dynein, light chain, roadblock-type 1, CACNA1C - calcium channel, voltage-dependent, l type, alpha 1c subunit); 2) Defense response to bacterium (S100A7 - s100 calcium binding protein a7, RIPK2 - receptor-interacting serine-threonine kinase 2, TLR3 - toll-like receptor 3, LYZ - lysozyme (renal amyloidosis), LTF - lactotransferrin); 3) Positive regulation of defense response (TLR8 - toll-like receptor 8, RIPK2 - receptor-interacting serine-threonine kinase 2, C3 - complement component 3, TLR3 - toll-like receptor 3, FABP4 - fatty acid binding protein 4, adipocyte); and 4) Epidermis development (S100A7 - s100 calcium binding protein a7, KRT34 - keratin 34, DSP - desmoplakin, SPRR2E - small proline-rich protein 2e, KRT5 - keratin 5, KRT83 - keratin 83, KRT31 - keratin 31).

The analysis for enriched GO terms performed on the set of DE genes separate for each time point (i.e., 40, 72 and 66 DE genes at T0, T1, and T2, respectively) revealed the following GO terms as the most significantly enriched: At T0, Defence response to bacterium (P = 1.77E-4) with 3 DE genes (LYZ, LTF and RIPK2); At T1, Intermediate filament (P = 4.75E-8) with 11 DE genes (KRT31, KRT33A, KRT33B, KRT34, KRT71, KRT83, KRT86, KRTAP3-1, KRTAP9-3, KRTAP9-4, and KRTAP13-3); At T2, Regulation of interferon-alpha biosynthetic process (P = 4.15E-4) with 2 DE genes (TLR3 and TLR8). The finding of keratin proteins over-represented at T1 and, by and large up-regulated among SUS sheep (Figure 3 and Table S1), indicates that the healing process begins immediately after the skin is damaged during the FR induction.

A subset of 10 genes from the list of DE genes was selected for validation using RT-PCR. Table 1 shows the least-square means resulting from the ANOVA analysis of the threshold cycles (C_t_) of each gene for each of the three sampling times in both the RES and SUS animals. Importantly, and in order to add confidence to the RT-PCR results, these animals were different to those used during the microarray analysis but were subjected to the same wetting regime.

**Table 1:** qRT-PCR Results: Goodness of fit as measured by the percent of the variation (R^2^) in threshold cycles (C_t_) explained by the ANOVA^1 ^model and C_t _least-squares means^2 ^for 10 DE genes across two genotypic lines (Resistant and Susceptible) and three time points (T0, T1, T2).

Gene	**R**^**2**^**,%**	**Least-Squares Means, C**_**t**_					
		Resistant			Susceptible		
		
		T0	T1	T2	T0	T1	T2
ABCC11	43.7	27.7^a^	29.3^b^	25.8^c^	27.1^a,c^	27.0^a,c^	26.5^a,c^
FABP4	88.8	32.6^a^	32.9^a^	30.5^b^	26.8^c^	26.3^c^	26.9^c^
FADS1	50.7	23.6^a^	23.14^a^	22.0^b^	23.9^a^	24.4^a^	22.4^a,b^
HMGCR	57.3	25.4^a^	27.4^a^	25.2^a^	26.6^a^	31.9^b^	20.5^c^
KLK10	47.6	25.8^a^	28.6^b^	27.5^a^	25.9^a^	28.0^a,b^	21.3^c^
KRT5	80.2	24.2^a^	32.0^b^	26.6^a^	21.8^c^	22.6^c^	18.1^d^
LYZ	61.5	33.3^a^	34.7^a^	34.6^a^	30.4^b^	33.1^a,b^	24.3^c^
S100A7	67.1	35.6^a,b^	35.3^b^	37.1^a^	32.1^c^	31.1^c,d^	29.3^c^
SPARC	55.2	18.8^a^	21.0^b^	19.7^a^	20.6^a^	23.0^c^	16.8^d^
ZMYM2	52.0	30.2^a,b^	32.6^a^	32.4^a^	29.3^b^	33.8^c^	23.7^d^

^1^We fitted an overall ANOVA model to obtain the least-square means of each gene and ten gene-specific ANOVA models to compute the R^2^. See Materials and Methods for details.

^2^Within a row, least-square means with different superscripts are statistically different at P < 0.05 significance level.

### Selection of candidate genes for fleece rot association studies

The list of 155 DE genes, along with the information from the RT-PCR results was then used to inform selection of candidates for genetic association studies. Genes were not selected on the basis of extreme DE alone. Instead, we developed a selection criterion that accounted for a biological function relevant to skin integrity, genes that are present in the overrepresented biological processes, genes from each of the three super-clusters and genes that were DE at the T0 time, as these are likely to prove the basis for the initiation of a rapid and effective immune response.

In total, 16 SNPs in the genomic regions of five candidate and DE genes (ABCC11, FABP4, FADS1, FBLN1, and HMGCR) were targeted for association studies. For each SNP, Table 2 presents the number of individuals genotyped, minor allele frequency (MAF), and P-value (P) against the test for Hardy-Weinberg equilibrium across the three populations (Armidale, Trangie RES line, and Trangie SUS line). While all markers showed a MAF > 1%, there was a large variation in the success of the genotype assay ranging from 16 animals from the Trangie SUS line for SNP FBLIn100090, to 233 animals from the Trangie RES line for SNP ABCex0667. A further SNP (FABIn20115) was found to be monomorphic for all individuals in the Trangie RES line.

**Table 2:** Number of individuals genotyped (n), minor allele frequency (MAF), and P-value (P) against the test for Hardy-Weinberg equilibrium for the 16 SNP used in the association studies across three populations.

SNP	Armidale			Trangie Susceptible			Trangie Resistant		
	
	n	MAF	P	n	MAF	P	n	MAF	P
ABCIn0150	69	0.43	0.633	137	0.45	1.000	149	0.19	0.290
ABCIn0270	71	0.31	0.583	81	0.27	0.009	80	0.39	0.000
ABCex0667	128	0.17	0.764	196	0.26	1.000	233	0.40	0.344
FABIn20115	147	0.22	0.002	n/a	n/a	n/a	n/a	n/a	n/a
FABIn20237	153	0.31	0.347	150	0.38	0.058	192	0.44	0.029
FABIn30227	148	0.23	0.009	156	0.02	1.000	161	0.01	1.000
FABIn30360	172	0.33	0.607	182	0.38	0.000	230	0.46	0.002
FABIn30420	147	0.10	0.362	184	0.02	1.000	207	0.07	1.000
FAD1g20645	145	0.28	0.000	150	0.13	0.133	167	0.06	1.000
FBLIn100090	58	0.37	0.006	16	0.34	0.093	26	0.23	0.280
FBLIn120135	119	0.36	0.031	161	0.40	0.000	162	0.46	0.003
FBLIn120280	148	0.40	0.002	160	0.45	0.017	215	0.37	0.660
FBLIn120995	137	0.48	0.004	124	0.50	0.000	122	0.48	0.000
FBLs10075	136	0.32	0.435	116	0.42	0.571	133	0.35	0.705
HMGIn40390	187	0.38	0.000	196	0.43	0.189	230	0.40	0.003
HMGIn60110	117	0.46	0.000	198	0.20	0.007	208	0.31	0.000

### SNP association to fleece rot

The results from the SNP association studies are shown in Tables 3, 4 and 5. In the Armidale pre-wetting trial, the SNP marker HMGIn60110 for HMGCR (3-hydroxy-3-methylglutaryl-coenzyme A reductase) gene was found to be significantly associated with FR score (P < 0.05) explaining 4.9% of phenotypic variance (Table 3). The regression coefficient associated with this marker indicates that selecting animals with allele T (i.e. genotypes 1, CT, or 2, TT) would result in a reduction of FR score by 0.21 units. However, this same SNP did not show any significant effect on FR score in post-wetting trial (Table 4), nor when the fleece rot score difference between pre- and post-wetting trials was considered (Tables 5).

**Table 3:** SNP association results for the pre-wetting trials in three populations.

SNP	Armidale			Trangie Susceptible			Trangie Resistant		
	
	ß	R^2^	P	ß	R^2^	P	ß	R^2^	P
ABCIn0150	-0.12	2.31	0.212	-0.03	0.04	0.810	0.05	0.40	0.442
ABCIn0270	-0.13	2.68	0.172	0.27	3.26	0.107	0.02	0.70	0.461
ABCex0667	0.18	2.79	0.059	0.00	0.00	0.966	-0.01	0.04	0.759
FABIn20115	0.09	0.68	0.322	n/a	n/a	n/a	n/a	n/a	n/a
FABIn20237	-0.08	0.91	0.240	0.11	0.58	0.352	**-0.09**	**2.23**	**0.039**
FABIn30227	-0.07	0.52	0.386	0.02	0.00	0.955	**-0.51**	**2.40**	**0.050**
FABIn30360	0.08	0.91	0.213	-0.10	0.42	0.382	**0.09**	**2.36**	**0.020**
FABIn30420	-0.11	0.64	0.336	0.15	0.10	0.677	-0.01	0.00	0.945
FAD1g20645	0.04	0.14	0.651	-0.08	0.12	0.672	0.05	0.15	0.624
FBLIn100090	-0.09	0.97	0.461	-0.17	2.31	0.574	0.03	5.96	0.229
FBLIn120135	-0.04	0.20	0.631	-0.03	0.03	0.820	**-0.17**	**6.75**	**0.001**
FBLIn120280	0.04	0.24	0.558	-0.10	0.46	0.393	**0.08**	**2.07**	**0.035**
FBLIn120995	-0.03	0.15	0.657	0.06	0.10	0.733	**0.17**	**6.22**	**0.006**
FBLs10075	-0.01	0.02	0.884	0.02	0.02	0.876	**-0.13**	**4.05**	**0.020**
HMGIn40390	0.05	0.26	0.487	0.01	0.00	0.953	0.04	0.50	0.287
HMGIn60110	**-0.21**	**4.89**	**0.017**	-0.06	0.13	0.620	-0.02	0.05	0.757

Statistics are as follows: ß: Regression coefficient of fleece rot score on genotype (indicating the average change in fleece rot score for each copy of an allele); R^2^: Percentage of phenotypic variation explained by the SNP (%); P-value: Probability value associated with the test ß = 0. Statistics corresponding to significant associations are highlighted in bold type.

**Table 4:** SNP association results for the post-wetting trials in three populations.

SNP	Armidale			Trangie Susceptible			Trangie Resistant		
	
	ß	R^2^	P	ß	R^2^	P	ß	R^2^	P
ABCIn0150	-0.10	0.23	0.696	0.01	0.02	0.882	-0.02	0.02	0.873
ABCIn0270	-0.35	3.00	0.149	0.15	1.89	0.222	0.05	0.05	0.838
ABCex0667	0.23	0.64	0.369	-0.09	0.56	0.295	0.06	0.19	0.505
FABIn20115	-0.13	0.20	0.594	n/a	n/a	n/a	n/a	n/a	n/a
FABIn20237	0.13	0.34	0.475	-0.15	1.55	0.129	0.01	0.01	0.919
FABIn30227	0.15	0.28	0.522	-0.21	0.25	0.539	0.00	0.00	0.999
FABIn30360	-0.11	0.22	0.543	0.18	1.97	0.059	-0.02	0.03	0.804
FABIn30420	0.14	0.13	0.659	-0.30	0.62	0.287	0.06	0.07	0.705
FAD1g20645	0.23	0.57	0.368	-0.18	1.17	0.187	-0.24	0.82	0.245
FBLIn100090	0.19	0.45	0.615	-0.13	1.09	0.700	-0.05	0.10	0.879
FBLIn120135	-0.19	0.60	0.403	0.00	0.00	0.965	-0.21	2.35	0.052
FBLIn120280	0.02	0.01	0.926	-0.08	0.43	0.409	0.10	0.58	0.266
FBLIn120995	**0.50**	**4.55**	**0.012**	0.09	0.30	0.547	0.10	0.50	0.438
FBLs10075	0.06	0.10	0.722	0.02	0.03	0.844	**-0.22**	**3.05**	**0.045**
HMGIn40390	0.01	0.00	0.947	-0.11	0.93	0.180	0.03	0.06	0.714
HMGIn60110	-0.22	0.62	0.401	-0.01	0.01	0.907	0.10	0.38	0.376

Statistics are as follows: ß: Regression coefficient of fleece rot score on genotype (indicating the average change in fleece rot score for each copy of an allele); R^2^: Percentage of phenotypic variation explained by the SNP (%); P-value: Probability value associated with the test ß = 0. Statistics corresponding to significant associations are highlighted in bold type.

**Table 5:** SNP association results for the difference between the post-wetting and the pre-wetting trials in three populations.

SNP	Armidale			Trangie Susceptible			Trangie Resistant		
	
	ß	R^2^	P	ß	R^2^	P	ß	R^2^	P
ABCIn0150	-0.01	0.00	0.973	0.14	0.30	0.528	-0.03	0.03	0.841
ABCIn0270	-0.28	2.04	0.235	-0.32	1.32	0.307	0.00	0.00	0.993
ABCex0667	0.05	0.04	0.829	-0.22	0.61	0.276	0.09	0.32	0.388
FABIn20115	-0.21	0.58	0.361	n/a	n/a	n/a	n/a	n/a	n/a
FABIn20237	0.15	0.54	0.368	**-0.46**	**2.78**	**0.041**	0.09	0.40	0.386
FABIn30227	0.21	0.66	0.328	-0.22	0.05	0.781	0.44	0.33	0.472
FABIn30360	-0.13	0.40	0.409	**0.54**	**3.50**	**0.012**	-0.10	0.36	0.362
FABIn30420	0.21	0.36	0.472	-0.71	0.65	0.276	0.10	0.12	0.614
FAD1g20645	0.17	0.37	0.470	-0.14	0.12	0.676	-0.33	1.14	0.169
FBLIn100090	0.25	0.90	0.478	-0.32	1.22	0.684	-0.17	0.98	0.63
FBLIn120135	-0.19	0.72	0.359	0.23	0.54	0.353	-0.09	0.29	0.493
FBLIn120280	0.03	0.01	0.886	-0.12	0.18	0.596	0.04	0.06	0.711
FBLIn120995	**0.52**	**5.71**	**0.005**	-0.09	0.07	0.774	-0.06	0.10	0.729
FBLs10075	0.02	0.01	0.896	0.09	0.10	0.730	-0.14	0.87	0.285
HMGIn40390	-0.05	0.05	0.772	-0.18	0.46	0.343	0.02	0.01	0.861
HMGIn60110	0.00	0.00	0.985	0.03	0.01	0.906	0.11	0.36	0.387

Statistics are as follows: ß: Regression coefficient of fleece rot score on genotype (indicating the average change in fleece rot score for each copy of an allele); R^2^: Percentage of phenotypic variation explained by the SNP (%); P-value: Probability value associated with the test ß = 0. Statistics corresponding to significant associations are highlighted in bold type.

In the Trangie SUS line, there was no SNP marker identified in either pre-wetting or post-wetting trials that had a significant effect on FR (Tables 3 and 4). However, significant associations for two SNPs (FABIn20237 and FABIn30360) from the same gene FABP4 (fatty acid binding protein 4) were identified for the difference between Post- and Pre-wetting FR score (P < 0.05, Table 5). They explained from 2.8% to 3.5% of the phenotypic variance.

An equally compelling result was observed in the Trangie RES line. In the pre-wetting trial, seven SNP markers from two genes (three from the gene FABP4- fatty acid binding protein 4, and four from the gene FBLN1 - Fibulin) were found to be significantly associated with FR scores, with R^2 ^ranging from 2.0% to 6.8% (Table 3). For one of them (FBLs10075), the significant association was maintained in the post-wetting trial, showing a decreasing effect (by 0.22 unit) on FR score (P < 0.05, Table 4).

## Discussion and Conclusions

In this study, we provide the first report of gene expression in the skin of sheep before, during and after an induced FR challenge. At each time, gene expression responses were compared between RES and SUS populations of sheep and genes DE between these phenotypic extremes were identified. A subset of candidates was chosen from the list for a genetic association study based on biological relevance or gene ontology. Hence, our underlying hypothesis was that DE genes could harbor SNPs that are associated with the FR phenotype.

Microarray analysis identified 155 genes that were DE and the majority of these genes were only significantly DE at one time of the challenge regime. A single gene, Host Cell Factor C2 (HCFC2), was DE at all three stages of the trial. In every case this gene was expressed at higher levels in RES sheep. Little is known about the biological role of this factor, although it has been shown to form a transcriptional regulatory complex with the human herpes simplex virus protein, VP16, and the transcription factor, Oct-1 [14]. Herpes simplex, like FR, is a disease of the skin making this an interesting parallel. The capacity to influence transcriptional regulation identifies HCFC2 as an attractive candidate for ongoing studies into the ability to resist fleece rot.

Our initial prioritization of candidates from the DE list for incorporation into the genetic association study was based on a literature review. The limited body of evidence available associates FR resistance with various immune cell populations, specifically IgE and cytokines [9,15,16]. However, none of these factors were identified in the current study. Instead, we chose to focus on genes that may contribute to the physical barrier function of skin, as this is the interface of the host and bacterial interaction. Numerous keratins (the structural subunit of hair) and collagens (the principal protein of skin and connective tissues) are contained in the DE list but we chose to focus on Fibulin (FBLN1) for further study. The Fibulin proteins form bridges that stabilize the various components of the extracellular matrix [17] and as such contribute to the integrity of the physical barrier. In the context of infectious disease, FBLN1 was found to be expressed at higher levels in the bed of non healing ulcers compared to the bed of healing ulcers [18]. Here, we found that SNPs in the FBLN1 gene were associated with both Pre-wet and Post-wet FR score in the Trangie RES population.

Lipids are also vital to barrier function as the loss of waxes, and hydrophobicity in general, is thought to be a major contributing factor to the development of fleece rot as recently reviewed [12]. Three genes (ABCC11, FABP4 and FADS1), that play roles in lipid metabolism, were identified from our DE list. Members of the ATP-binding transport protein superfamily, including ABCC11, are involved in the transport of sphingolipids, glycerophospholipids, cholesterol and fatty acids in epidermal lipid reorganization during keratinocyte terminal differentiation [19]. A SNP in the ABCC11 has been found to be the determinant of ear wax type in humans [20] and, subsequently, axillary osmidrosis in humans [21]. In axillary osmidrosis, bacteria such as *Corynebacterium sp*. metabolise the ear wax, producing the symptomatic strong odour [21]. Interestingly, an earlier report from our group identified *Corynebacterium sp. *as the most abundant bacterial genera present in the fleece of SUS sheep [22]. However, no SNPs in this gene were associated with a fleece rot phenotype.

A second lipid metabolic gene resident within the DE list was fatty acid desaturase 1, FADS1. The proteins encoded by genes of the fatty acid desaturase (FADS) gene family are responsible for the production of arachidonic acid and eicosanoids from long-chain fatty acids (PUFAs). Recently, fatty acids have been suggested to play a role in the development of inflammatory disorders and allergies [23]. Genetic variants of the FADS1 - FADS2 gene cluster were also associated with the fatty acid composition of human serum and to have an impact on atopic diseases [24]. As was the case with ABCC11, no SNPs in FADS1 were associated with resistance to fleece rot.

In a second approach, the genes selected for the genetic association study were members of pathways deemed to be overrepresented in the list of DE genes by the gene ontology over-representation analysis. This analysis identified four biological processes whose members were over-represented among the list of DE genes. Two of the processes, 'defense response to bacterium' and 'positive regulation of defense response', are related to the initiation and functional performance of defense pathways. Given the bacterial nature of FR, it is reassuring to see such pathways associated with variation in responsiveness. Similarly, the involvement of genes associated with a third pathway 'epidermal development' is consistent with the likely damage caused to skin during FR infection and recovery of the epidermal barrier. The relationship of the fourth pathway 'visual behaviour' to fleece rot is less clear. This process is defined by the Gene Ontology Consortium as the actions or reactions of an organism in response to a visual stimulus [25].

The gene S100A7 was present on two of the biological processes. S100A7 is involved in the regulation of a cell cycle progression and differentiation and the protein is markedly over-expressed in the skin lesions of psoriatic patients, wound healing, skin cancer, inflammation and cellular stress. S100A7 gene is a member of the human 1q21 locus that is associated with atopic dermatitis [26]. Unfortunately we were unable to identify any SNP in this gene that were suitable for ongoing analysis. Further efforts may resolve this issue. Additionally, S100A7 has been reported to interact with the epidermal fatty acid binding protein (FABP5) where increased expression of S100A7 results in increased expression of FABP5 and vice versa. FABP5 and S100A7/FABP5 complex bind oleic acid suggesting a role in oleic acid transport and metabolism [27]. Oleic acid may have a role in inflammation as topical application modulates epidermal Langerhans cell density. The complex could also function in lipid metabolism and transport during epidermal barrier assembly and may also modulate epidermal inflammatory response in epidermal diseases.

Although FABP5 was not identified as DE in this study, a related fatty acid binding protein, FABP4, was identified. Fatty acid binding proteins are hydrophobic ligand binding cytoplasmic proteins and are thought to be involved in lipid metabolism by binding and intracellular transport of long-chain fatty acids. Studies also imply roles of FABP family proteins in cell signaling, inhibition of cell growth and cellular differentiation. FABP4 has been shown to be induced in Pten-null keratinocytes, suggesting a role in sebaceous gland differentiation [28]. Importantly, SNPs in FABP4 were associated with FR score in the Trangie RES line pre-wet and in the SUS line post-wet.

The gene 3-hydroxy-3-methylglutaryl-coenzyme a reductase or HMGCR is a member of the 'visual behaviour' grouping. Cholesterol synthesis is regulated by the rate-limiting enzyme HMG CoA reductase. Cholesterol is part of the epidermal surface lipid-based barriers and their role in a number of skin conditions has long been established in that a disturbed skin barrier is an important component in the pathogenesis of contact dermatitis, ichthyosis, psoriasis, and atopic dermatitis. Acute epidermal barrier disruption leads to an increase in HMGCR activity [29]. The activity of HMGR was reportedly increased following barrier disruption due to both an increased quantity of enzyme and an increase in activation state [30]. Notably, SNPs in this gene were associated with the prewet FR score in the Armidale flock.

Other pathway members that would be worthy of future study include the pathogen associated molecular pattern detection receptors TLR3, TLR8 and the RIPK2 signal transduction molecule. The presence of TLR3 in the DE list is intriguing, as this receptor is classically associated with a response to viral infection. The elevated expression of this receptor suggests a yet to be defined viral component that may opportunistically form part of the FR infective complex. Although skin is regarded as an unusual route of entry for viruses, the damaged surface caused by the bacterial component of the disease may leave sheep susceptible to later viral infection. Also the involvement of HCFC2, as described earlier implies that there is some circumstantial evidence that viruses that do preferentially target skin, such as herpes, may be involved.

The microarray component of this study has identified a number of genes that are likely to contribute to an ability to resist FR development. Expression results were then used to inform a selection of candidates for genetic marker association with the FR phenotype. Gene association studies were performed in a population of animals independent of those in which the microarray studies were performed. As a result, we have identified FBLN1 and FABP4 as key factors in this response. Validation of these markers in other populations could lead to vital tests for marker assisted selection that will ultimately increase the natural fleece rot resistance of sheep.

## Methods

### Sheep resources, fleece rot induction trial and skin biopsies

All experimental work was conducted according to ethical procedures approved by the Industry and Investment NSW, Animal Ethics Committee (Approval Number 03/011), and the CSIRO Livestock Industries, FD McMaster Laboratory, Animal Ethics Committee (Approval Number 03/71).

Twenty FR and fly strike RES and 20 SUS sheep were supplied by Industry and Investment NSW, Agricultural Research Centre (Trangie, NSW, Australia). Briefly, these populations of sheep have been divergently selected from a founder population of medium wool Peppin Merino sheep since 1978, on the basis of natural and experimentally induced fleece rot and natural fly strike [4,31,32]. Additionally, a population of 239 outbred adult Merino × Romney cross ewes, from the CSIRO mapping flock (Armidale, NSW, Australia), was also included in this experiment [33]. All sheep were 14 months of age and female.

Induction of FR occurred in a modified animal house, accommodating ~100 animals at any one time. This facility, known as the 'wetting shed', allowed precise control of simulated rainfall. As such, the procedure for FR induction is standardised. Prior to entering the shed, animals were given *ad lib. *access to the diet for at least 7 days. Animals were randomly allocated to pens (a maximum of ~50 animals per pen) within the shed and fed a pelleted lucerne mix *ad lib*. After this initial adjustment period, the animals were subjected to simulated rainfall from overhead sprinklers that mimic rainfall at the rate of about 1 mm/min. A standard induction treatment mimics 9 -10 mm of rainfall per day by 2 hourly showers of 45 second duration. Animals remained in the shed for 5 days during FR induction, then for 24 hours at the start of the drying phase. Animals then were removed from the shed. During this period, animals were kept dry (ie., not exposed to natural rainfall) and monitored for occurrence of flystrike (as animals are more susceptible at this time).

A standardised system, described by Raadsma *et al. *[31], was used to score fleece rot incidence and severity independently of the incidence of flystrike (occurring naturally or induced). Scores of 0 to 5 for fleece rot severity were given as follows: 0 = No bacterial colour or crusting; 1 = Band of bacterial staining < 10 mm wide with no crusting; 2 = Band of bacterial staining > 10 mm wide with no crusting; 3 = Band of crusting < 5 mm wide with or without bacterial staining; 4 = Band of crusting from 5 to 10 mm wide with or without bacterial staining; 5 = Band of crusting > 10 mm wide with or without bacterial staining. Each animal was scored at four sites by parting the fleece along the animal's backline (back of neck, wither, loin and rump). The highest score at any one site was the final fleece rot score given and was a measure of the animal's overall susceptibility. All animals within a flock (Armidale or Trangie) were scored by a single technician.

**Figure 4:**
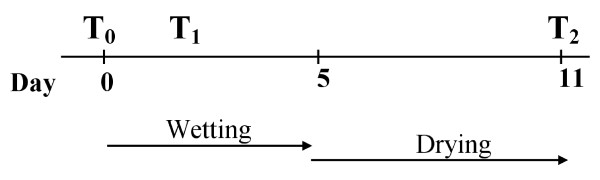
**Schematic of fleece rot induction and skin biopsies**. The first skin biopsies were taken on the first day (T0) prior to commencement of the five day regime of simulated rainfall. Subsequent skin biopsies were taken 2-3 days into the wetting regime (T1) and following a recovery period of 11 days from the time that the wetting regime had begun (T2).

Two separate challenge trials were performed as follows: First, because no fleece rot susceptibility data was available for the CSIRO mapping flock, all 239 sheep were initially put through a fleece rot inducing wetting trial, so they could be scored and sorted into RES (low score) and SUS (high score) groups; Secondly, 40 ewes (20 RES and 20 SUS) were selected from each of the Trangie selection flock and the CSIRO mapping flock and subjected to a wetting trial. Figure 4 provides a flow diagram of the challenge trial including the time points when FR measurements and skin biopsies were taken as follows: The first skin biopsies were taken on the first day (T0) prior to commencement of the five day regime of simulated rainfall. Subsequent skin biopsies were taken 2-3 days into the wetting regime (T1), and following a recovery period of 11 days from the time that the wetting regime had begun (T2).

For skin biopsies, the midline region over the shoulder was selected, and a wool staple sample was removed by close clipping a region of approximately 5 cm^2^. Two skin trephines of 0.9 cm diameter were taken from the clipped region and immediately placed in RNA later (Ambion) for subsequent RNA extraction. Skin samples in RNA later were placed into -80°C freezers for long-term storage.

After all FR measurements and skin biopsies were taken, each group of 20 sheep was split into three sub-categories based on the rank of their post-wetting (T1) FR scores. The designations of these sub-categories for the resistant groups were highly resistant (RH), average resistance and low resistance (RL). For susceptible groups the sub categories were highly susceptible (SH), average susceptibility and low susceptibility (SL). Overall, RH animals had a FR score of 0 to 1, while SH animals had a score of 4 to 5. Representative RL and SL animals had FR scores of 2 to 3 at T1.

### Construction of subtracted cDNA libraries

RNA from two RES (one RH and one RL) and two SUS (one SH and one SL) animals from the Trangie flock was used in the construction of six subtracted cDNA libraries in a layout designed to maximise the chances to capture the cDNA clones responsible for the RES and SUS differences within and across time points as follows: 1) SL at T1 subtracted from RL at T0; 2) RH at T1 subtracted from SH at T1; 3) SL at T0 subtracted from RL at T1; 4) SL at T1 subtracted from RL at T0; 5) RL at T1 subtracted from RH at T2; and 6) SH at T2 subtracted from SL at T1.

Total RNA was prepared from the adult Merino skin samples using TRI Reagent^® ^http://www.mrcgene.com/tri.htm in accordance with the manufacturer's recommendations (Sigma, St Louis, MO, USA). In each extraction, 5 mL of TRI Reagent was used to extract total RNA from 0.5 g of skin sample. Total RNA was treated with DNase I (Ambion DNA-free™, Austin, TX, USA) to minimise the presence of genomic DNA. mRNA was purified from each sample using GenElute mRNA mini preparation kit (Sigma). Total RNA and mRNA were quantified by spectrophotometric measurements at 260 nm and 280 nm. Purity was verified by OD_260_/OD_280 _ratio > 1.8. cDNA was cloned into the pGEM-T Easy plasmid (Promega) and cultured in OmniMAX™ 2-T1R *E. coli *cells (Invitrogen). Approximately 960 colonies were picked from each library.

To investigate the quality of libraries and the identity of the clones, 100 random clones from each of the six libraries were sequenced using M13 universal forward primer and ABI Prism^® ^BigDye terminator sequencing mix 3.1 (Applied Biosystems, USA). Clone inserts were annotated based on sequence similarity identified by BLASTN and BLASTX [34] in the GenBank non-redundant and human and bovine reference sequence data sets at National Centre for Biotechnology Information (NCBI). A cut-off score of 70 or better (E-value of 10^-10 ^or better) was used to assess the significance of sequence alignment. Functional annotations were derived from the gene ontology consortium [35]. Assignments for each of the genes were subsequently conducted using the Entrez Gene [36] data sets at NCBI. Across the six subtracted and normalised FR cDNA libraries, 445 total unique sequences were obtained from 600 clone sequences. Therefore, the level of redundancy was calculated as 23% by comparing the number of unique sequences with the total number of clones sequenced.

### Microrray preparation

A combined Ovine-Bovine cDNA microarray was produced. Ovine cDNA clones consisted of ~960 anonymous cDNA clones from each of the six libraries (totalling 5,760 clones) and a subset of ~2,300 ESTs (6 × 384-well plates) selected from foetal and adult sheep skin libraries [37]. The foetal and adult sheep skin libraries sequences are amongst those deposited in GenBank with Accession Nos. CF115819-CF118833. Bovine cDNA clones consisted of ~4,200 ESTs (11 × 384-well plates) prepared from adult bovine skin [38]. Bovine sequences are amongst those deposited in GenBank with Accession Nos. CF762013-CF769317. The ovine FR cDNAs in pGEM-T Easy were amplified by PCR in a 65 μL reaction containing 60 μL PCR master mix (50 μM dNTP, 0.15 μM forward primer, 0.15 μM reverse primer, 10 mM Tris-HCl pH 8.3, 50 mM KCl, 1.5 mM MgCl_2 _and 0.6 U of Taq F2 DNA polymerase Fisher Biotech) and 5 μl cDNA template in 96 well plates. The ovine skin pTriplEx cDNAs were amplified by PCR in a 70 μL reaction volume in 96 well plates containing 1 μL plasmid DNA template, 15 pmol pTriplEx 5' and 3' sequencing primers, 0.2 mmol/L dNTPs, 3.0 mmol/L MgCl_2_, and 1.4 units Fisher Biotech Taq polymerase. The bovine cDNAs were amplified using M13 universal forward and reverse primers and 1 μL phage DNA template. Amplified DNA was purified using a standard isopropanol precipitation procedure. Before spotting on glass microarray slides, amplicons were resuspended in water and transferred into 31 × 384-well plates.

A total of 11,689 probes were printed in duplicate onto Corning UltraGAPS (Corning Inc., NY, USA) glass slides using a BioRobotics MicroGrid II TAS and BioRobotics 2500 pins (Genomic Solutions, Ann Arbor, MI, USA.) at a spacing of 210 μm. Elements were printed on the array arranged in 48 blocks of 23 rows by 22 columns each. DNA was covalently crosslinked by baking at 80°C for 2 hr.

### Total RNA preparation, labelling and array hybridisation

Total RNA was prepared from the skin samples using TRI Reagent in accordance with the manufacturer's recommendations (Sigma, St Louis, MO, USA). In each extraction, 5 mL of TRI Reagent was used to extract total RNA from one trephine of skin. Total RNA was treated with DNase I (Ambion DNA-free™, Austin, TX USA) to minimise the presence of genomic DNA. RNA quality was assessed using agarose gel electrophoresis and quantified by spectrophotometry.

**Figure 5:**
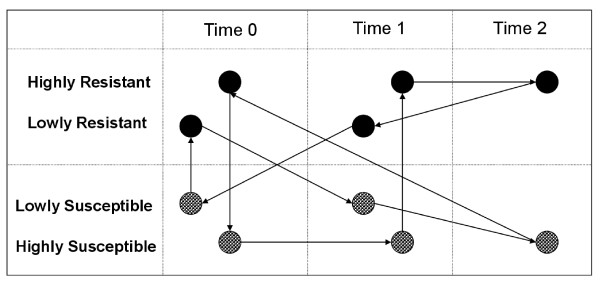
**General microarray hybridisation loop design**. The design configuration is a loop design that compares the high and low resistant animals to the high and low susceptible animals within and across time points of fleece rot induction (T0-T1) and recovery (T2). Arrows indicate hybridisations and go from the samples labelled with red (Cy5) to the samples labelled with green (Cy3) dye.

The experimental layout of the microarray hybridisation design (Figure 5) enabled each sample of total RNA to be labelled only once with each of Cy-3 (green) and Cy-5 (red) and required a minimum of ~34 μg total RNA. For samples that yielded enough total RNA for each Cy-3/Cy-5 pair, labelled cDNAs were purified to remove unincorporated dyes using QIAquick PCR purification columns (QIAGEN) then dried to ~1.0 μl.

### Microarray experimental design and data acquisition criteria

The general experimental design for the microarray hybridisations is shown in Figure 5. The design took into account the limited animal material for each time point, cost of each array slide and hybridisation and was developed with an emphasis on the identification of early changes in gene expression profile between RES and SUS animals at T0, T1 and T2. The design layout corresponds to a circular alternate dye-swap loop configuration by which each RNA sample intervenes in two hybridisations, one red and one green. It also incorporated biological variation by repeating each hybridisation three times, each with a different set of animals. Hence, three biological replicates were employed at each time point, two from the Trangie Merino flock and one from the CSIRO Merino × Romney flock. In total, 31 hybridisations were performed including, as a measure of experimental noise, a self-self hybridisation performed with the RL sample taken at T0 for one of the Trangie set of biological replicates.

We used the GenePix 4000A optical scanner (Molecular Devices, Sunnyvale, CA, USA) and the GenePixPro 5.1 image analysis software (Molecular Devices) to quantify the gene expression level intensities. Criteria for data editing were as follows: First, probes with a signal to noise ratio less than one in all hybridisations were deemed undetectable and removed from the analysis (10,094 probes out of the original 11,689 passed this criterion); Second, for genes represented by more than one probe, the most abundant probe, averaged across all hybridisations, was assigned to that gene. This second criterion is based on the fact that abundant probes are better annotated and their intensity signals less prone to noise. These resulted in 409,324 gene expression intensity readings (half from each colour channel and 13,204 from each chip) 3,238 unique skin-specific genes being included in the analysis. Prior to normalization, expression intensity readings were background corrected and base-2 log-transformed. The arithmetic mean and standard deviation (in brackets) for the red and green intensities were 7.95 (2.99) and 8.53 (2.21), respectively.

The expression data from the entire set of 31 hybridisations was deposited in Gene Expression Omnibus (GEO; http://www.ncbi.nlm.nih.gov/geo/) and can be downloaded and can be accessed using accession number GSE21022.

### Data normalization and identification of DE genes

An ANOVA mixed-effect model was employed to normalize the data as previously described [39]. In detail, gene expression data normalization was achieved by fitting the following ANOVA mixed-effect model:

where Y_*ijkftmn *_represents the *n*-th background-adjusted, base-2 log-intensity from the *m*-th gene at the *t*-th treatment (time point and breed line) taken from a sheep from the *f*-th flock, from the *i*-th array, *j*-th printing block and *k*-th dye channel; μ is the overall mean; C represents a comparison group fixed effect defined as those intensity measurements that originate from the same array slide, printing block and dye channel; G represents the random gene effects with 3,238 levels; AG, DG, FG and TG are the random interaction effects of array × gene, dye × gene, flock × gene, and treatment × gene, respectively; and e is the random error term.

In this notation, treatment was defined as the combined effect of breed line (RES and SUS) at the three time points (T0, T1 and T2). Using standard stochastic assumptions, the effects of G, AG, DG, FG, TG and e were assumed to be independent realizations from a normal distribution with zero mean and between-gene, between-gene within-array, between-gene within-dye, between-gene within-flock, between-gene within-treatment and within-gene components of variance, respectively. Restricted maximum likelihood (REML) estimates of variance components and solutions to model effects were obtained using the analytical gradients option of VCE6 software ftp://ftp.tzv.fal.de/pub/vce6/.

The solutions to the TG effect were used as the normalized mean expression of each gene in each of the conditions under scrutiny (breed line and time point). Finally, the difference between the normalized mean expression of a gene in the two breed lines at each of the three time points was computed as the measure of (possible) differential expression. Three measures of DE were explored, each across the two breed lines and within time points. In order to emphasize the larger information content expected in the high lines of each breed line, twice as much weight was given their normalized solutions.

In algebraical terms, the contrasts corresponding to the three measures of normalized DE were as follows:

Where RH0, RH1 and RH2 correspond to the highly resistant line at times T0, T1 and T2, respectively; SH0, SH1 and SH2, correspond to the highly susceptible line at times T0, T1 and T2, respectively; RL0 and RL1 correspond to the lowly resistant line at times T0 and T1, respectively; Finally, normalized DE measures beyond 2.58 standard deviations from the mean were deemed to be significantly different from zero at P < 0.01.

Hierarchical clustering of DE genes was performed using the PermutMatrix software [40] and Gene Ontology terms over-represented among the DE genes were identified using the GOrilla tool [41] using the list 3,238 genes included in the analysis as the background list in the over-representation analysis.

### qRT-PCR validation of DE genes

The expression patterns of 10 selected transcripts DE according to microarray analysis were further examined using quantitative real-time PCR (qRT-PCR) on five animals from the resistant line (all with FR score of zero) and three mid-range animals from susceptible line (all with FR score of 3). Hence, samples used in the qRT-PCR were from different animals to those used in the microarray experiments. Table 6 lists the gene-specific primer pairs that were designed and used in the qRT-PCR.

**Table 6:** Sequences (5'-3') of forward and reverse primers used in the real-time PCR.

Gene	Forward primer sequence	Reverse primer sequence
ABCC11	CAAGTTCTCGGTTATCCCTCAA	AGAAGTTTGAGCCATTTTCCAC
FABP4	TGAAATCACTCCAGATGACAGG	TCAATATCCCTTGGCTTATGCT
FADS1	GACCGAAAGGTGTACAACATCA	ATTCTTAGTGGGCTCAAAGCTG
HMGCR	TAGAGGCACAGGAACCTGAAAT	GGCGAATAGATACACCTCGTTC
KLK10	CCATGCACACCTGCTAACAT	CTTGCCCAAGGTCACACAG
KRT5	AGGAGGCTCCATTTGGTCTC	AAGAGGTCACCGTCAACCAG
LYZ	ACTCTGAAGAGACTCGGATTGG	GTTAACAGCTCTTGGGGTTTTG
S100A7	TGACATCTCCTCTGATCAGCTC	CAAGTATTGTCTGCCCCTTTTC
SPARC	CTTGCCTGATGAGACAGAAGTG	GTGTTGTTCTCGTCCAGTTCG
ZMYM2	TTTTTCCAGTGCCTAAACACAGT	AGCATACTTCCAGACGGGTCA

The qRT-PCR was performed using the SYBR Green system in an ABI Prism 7900 Sequence Detection System (PE Applied Biosystems, Foster City, CA). Measurement of relative gene expression for each candidate DE gene was conducted with the reference gene testis enhanced gene transcript (TEGT) chosen on the basis of its moderate and consistent expression in the microarray analysis. Quantitative PCR amplification efficiency was calculated from the slope of a standard curve for each gene of interest using a ten fold dilution series of standard pooled skin cDNAs as template. All candidate genes qPCR amplification efficiencies were between 1.8 and 2.2. Finally, each PCR was conducted in quadruplicate.

For the analysis of the qRT-PCR data the procedure GLM of SAS 9.1 (SAS Institute Inc., Cary, NC, USA) was employed to fit an ANOVA model to the threshold cycles (C_t_) resulting from each PCR reaction. An overall model was fitted that contained the effects of gene, genotypic line (RES or SUS), time point (T0, T1, T2) animal nested within line, PCR plate, the three-way interaction of gene × line × time and residual. This overall model was used to compute and test the significance of the least-squares means of C_t _for each gene. Subsequently, a gene-specific ANOVA model was fitted after removing the gene effect from the overall model. These gene-specific models were employed to ascertain the goodness of fit (R^2^) of each model as measured by the percent of variation in the C_t _of a given gene that is explained by the model. The expectation being that higher R^2 ^values should result for DE genes.

### Primer design, PCR and sequencing for SNP identification

Using information from the bovine genome sequence and available ovine public database sequences, between 3-5 primer sets amplifying either intron or exon regions were designed for a total of ten candidate DE genes (Table 6). Multiple primer sets were tested on genomic DNA and those that amplified the correct products were then used for amplification of sheep genomic DNA from six RES and six SUS sheep from the Trangie flock. PCR products were sequenced and compared using Sequencher v4.2.

In order to validate the SNPs, further PCR and fragment sequencing were conducted using 12 Trangie animals and 12 (six SUS and six RES) Merino sheep from the CSIRO AB78 Mapping Flock.

In order to address the need for high throughput genotyping of animals and cost effectiveness of the process, a SNPlex assay was then designed. The SNPlex assay enables the simultaneous genotyping of multiple SNPs against a single sample. The initial criteria applied to assess the suitability of a SNP to be included in the SNPlex multiplex primer sets was that a SNP must have a minimum of 50 bp of good quality sequence either side of its sequence and with preferably no other SNP within this sequence. This resulted in a total of 16 SNPs from five target genes being successfully incorporated into an assay which was later used to genotype the two resource flock animals. Table S2 (Additional file 2) provides the sequence for the 16 SNPs used in this study including the results from the sequence alignment (BLAST) analyses. Not surprisingly, the most accurate hit often corresponded to the Bovine genome.

### SNP association to fleece rot

Both the Trangie flocks and the CSIRO AB78 mapping flock (Armidale) were used for the SNP association study. In total 581 Merino animals from the Trangie RES and SUS flocks and a sub-set of DNA samples comprising 206 animals from the Armidale flock 1997 drop were genotyped against 16 SNPs. Due to the nature of Trangie animals coming from different selection lines, the SNP association analyses were performed separately for Trangie SUS line (270 animals), Trangie RES line (311 animals) and CSIRO Armidale populations. In all populations, FR measurements were recorded in pre-wetting and post-wetting trials. FR scores at pre-wetting, post-wetting and the difference (pre-wetting minus post-wetting) were analysed.

Prior to the SNP association study, preliminary analyses were carried out on FR scores using a mixed animal model (ASreml_R2.0) to test for significant environmental effects and polygenic component of inheritance. The statistical model included fixed effects of flock, sex, DOB (date of birth), birth type, rear type and a random effect of animals (taking pedigree relationship into account). DOB was treated as a covariate. The residual values from the model were then combined with SNP genotype information for further SNP association evaluation.

For each individual SNP, a Hardy-Weinberg equilibrium (HWE) test using Fisher's Exact Test Statistic was conducted to check whether the observed three genotypes (e.g. CC, CT and TT) of the SNP conformed to Hardy-Weinberg expectations. An additive model was then fitted in which fleece rot scores for heterozygous individuals were assumed to be midway between the values of animals having alternative homozygous genotypes. This analysis was a simple linear regression of FR score on the number of copies of one allele of a SNP present in each individual (corresponding to genotypes 0, 1 and 2) [42]. The regression coefficient derived from the analysis corresponded to an additive effect of a SNP allelic substation effect, i.e., average change in fleece rot score for each copy of an allele. The effect of a SNP was also represented in terms of the percentage of phenotypic variance it explained (R^2^).

## Authors' contributions

WS carried out the animal studies, conducted the laboratory experiments, tabulated the data and drafted the manuscript. YL performed the SNP association analyses and drafted the manuscript. AI contributed to review and writing the manuscript. EC assisted in the SNP genotyping. SMcW performed the bioinformatics, sequence annotation and GEO submission. TD carried out the animal studies and assisted in the laboratory experiments. RM contributed to the design and printing and of the microarray slides and reviewed the manuscript. BN, SM and AR conceived the study, participated in its design and coordination and contributed to review and writing the manuscript. All authors have read and approved the final manuscript.

## Supplementary Material

Additional file 1 **Differentially expressed genes **Normalized mean expression for the 155 differentially expressed genes across the 6 conditions (2 lines, resistant and susceptible, by 3 time points) Click here for file

Additional file 2 **Annotation of SNPs**. Sequence annotation of the 16 SNPs used in the genotyping program. Click here for file
