# Supplementary material for: A genomics-informed, SNP association study reveals FBLN1 and FABP4 as contributing to resistance to fleece rot in Australian Merino sheep
Source: BMC Vet Res. 2010 May 26;6:27. doi: 10.1186/1746-6148-6-27 (PMC2886023; doi:10.1186/1746-6148-6-27)
Supplement: Additional file 2 — Annotation of SNPs. Sequence annotation of the 16 SNPs used in the genotyping program. [file 1746-6148-6-27-S2.DOC]

**Table S2. Sequences and annotation results corresponding to the 16 SNPs used in this study.** The nucleotide position of the SNP is highlighted in bold type.

| **SNP** | **Sequence** | **BLAST (*Bos taurus genome)*** | | **Sequence ID**^A^ | | **E-value**^B^ |
| --- | --- | --- | --- | --- | --- | --- |
| ABCIn0150 | GGTGAGTAAGTGCAAGGCTGAATTCTCAGTCCTGCTGGTCCTCGGGGCTCCTCTGGTGTGGTATGCCAGAGGCTGCGTGGATTAAATCATCTCA**[C/T]**GGGAATGAGATGGTTTGAGATCCCTAGATAAAGGCTGATGATGCCAGTAGTAAGCCAGGCATGTACCAGCTCAGGGCAAGGGCTGTACAGCTTTG | Similar to ATP-binding cassette, sub-family C, member 11 | | NW_001493578.2 | | 2e-70 |
| ABCIn0270 | AGATAAAGGCTGATGATGCCAGTAGTAAGCCAGGCATGTACCAGCTCAGGGCAAGGGCTGTACAGCTTTGTCTTTCTGGTGCTTACAG**[C/T]**CAGGAGAGACTCAGATGGGACCAGTGCTGTGTAGGTATTGATAAGAGCTGCAGACACCGCAGTGGGTGCCCAGCCAGGGCCTCTGT | Similar to ATP-binding cassette, sub-family C, member 11 | | NW_001493578.2 | | 8e-68 |
| ABCex0667 | GGTGACATACTGGACAATCCTCAGCTGCCCTTTTACCAACTGGTGTTTGGCCTCAGCAGCCTGTTTGCAGTCTTCCTGGGCATCTTGCTCTCAGTGGTTTTCAC**[C/T]**AAGGTGATGGGGAAGGCATCCACAGCATTGCACAACAAGCTCTTCAACAA | Similar to ATP-binding cassette, sub-family C, member 11 | | NW_001493578.2 | | 1e-60 |
| FABIn20115 | ACATGTAATTTTGCATATTGTTTTTGGCATTCATTGTTTTCTTTTTCAACATTTTCTTGTAATTTAGAATTGCTAAGTA**[C/A]**CTCAAAATAAGCAAATAAAAGTGCTCTATTTTTTTCCCCCTCCATGATTGTAATCACTTTTAATTATCCCCACAGAGCATCATAAA | Fatty acid binding protein 4, adipocyte | | NW_001493222.2 | | 6e-24 |
| FABIn20237 | AAAAAAAAACACCTATGATGCTATTCCACATAAATTTATTATGTATATTCTTTCAC**[A/G]**GTATTTTTTCAAATGCATGTTTGTATAATATTCTGATCATAATATACATGTAATTTTGCATATTGTTTTTGGCATTCA | Fatty acid binding protein 4, adipocyte | | NW_001493222.2 | | 5e-49 |
| FABIn30227 | ATCGCCCTTGTAGAATGAAAAGTTAGTCTATTGGGATTATGGTTTCACTCTGGCAATTATCCTTCTAAGCTCTGTCTATGTATA**[C/T]**TGTGCCCCAGGAAGTATTTTCTTATCCCTCTCAATGTGAACCATATTGTATTGTGCATTTCTAATTATGTTTTTCATTCACCACA | Fatty acid binding protein 4, adipocyte | | NW_001493222.2 | | 1e-70 |
| FABIn30360 | CATTTCTAATTATGTTTTTCATTCACCACATAGATTGTAAGATTCCTTGAGGGCAAGACTTGTATCTTCTTGATCT**[C/T]**TGTGTCTCCCTAGTTTATTACAATATCAGGTATATAAGAAGAGCCAAGAGTGAATATCTTTTGATGAACATTTTTTCCTTCTC | Fatty acid binding protein 4, adipocyte | | NW_001493222.2 | | 1e-66 |
| FABIn30420 | GATTCCTTGAGGGCAAGACTTGTATCTTCTTGATCTTTGTGTCTCCCTAGTTTATTACAATATCAGGTATATAAGAAGAGCCAAGAGTGAATAT**[C/T]**TTTTGATGAACATTTTTTCCTTCTCAGCATTGAAGGAGACAATAAATAAATAAACCATGAGTTGTTTAGTCCTGAGGTTTTTACCAAATATTTTGCC | Fatty acid binding protein 4, adipocyte | | NW_001493222.2 | | 1e-56 |
| FAD1g20645 | CTTCGCTGACATTGTCCACTCACTGAAGGAGTCCGGGCAGCTCTGGCTAGATGCCTATCTCCACCAATAACAGCC**[A/G]**CCTCCCTCTGCAAGAAGGGTTATGGCACCAAAGCAGACGGAAGCTTGGGGCAATGCCACTACAATCCTAATATTCAGAGGGGG | Similar to fatty acid desaturase 1 | | NW_001494536.1 | | 1e-61 |
| FBLIn100090 | GACTTCCCCTGAAGTTTCCGCTGTGAGTGCAAGGCGGGTACTACTTCGACGGCATCAGCAGGACATGTGT**[G/A]**GGTATGTGTGGGTATCCCTGAGGCCGGGTGCTGGCCAGGTGACGCCAAGGAGAACCAGCCTGGGGTGCACCCTGGAGTG | Fibulin 1 | | NW_001495117.1 | | 3e-47 |
| FBLIn120135 | GAAGGTGAGCGGGGAGAGGCCGCCTCTCTCCTGTCGCCCACTCCCCGCTCCTCCTGGCTGGCGAGTGGCCC**[G/A]**GGTTTCTTCCCTCTGAGATGTGTTTGGATGGACTTTGCCCATTGTTGAAAGTGAGA:TTTGTAAAGGAGGAAACCCACTCCCAGA | Fibulin 1 | | NW_001495117.1 | | 3e-66 |
| FBLIn120280 | TTTGTAAAGGAGGAAACCCACTCCCAGATTTTCTTCAGTGACATAAAATGGAACGGTGGTGGTGGCTATGGGTGCCGCC**[C/T]**GGACCTCAAGCAGGGCGGGGGTGCGGGAGGNGGGCTGAGGAGTCACACCCTGGCTCGACCCTCTGCCTCTGGC | Fibulin 1 | | NW_001495117.1 | | 6e-54 |
| FBLIn120995 | GAGAACCCCCTGTGTCCYGCTCATCCTCGGGGCTGACTTCCAGGTCACAGCTCAGCTC**[C/A]**CTGGGGGATGTAGCCCAGCGAGGGGGCCCAACGCAGATGCTGAGGGTAGGTTTTGCC | No significant similarity found in NCBI NR database | | | | |
| FBLs10075 | GGAACTGGCTATGAGCTCACAGAGGATAATGACTGCAAAGGTACGACAGGCTCCGGGT**[C/T]**GCAGAACCTCACCCCCGGGAGTAGATGCGATGTGATGAGAAAGAGGCTGGGGGGGTCCAGGGCTCCACGTGCTGGGTCCCCCAG | Fibulin 1 | NM_001098029 | | 3e-09 | |
| HMGIn40390 | CTACCACTCACTGACACAAGTTCTTAAGTTCCAAATTTTTTGAGATCATGTTAGTATTATGAAACTTAAATAT**[C/G]**CTTGTCAAAATGCTCAAATGATCATAAATAAATATTCATCAAATACATGCTCAGGGCAGAAGTA | 3-hydroxy-3-methylglutaryl-coenzyme A reductase | NW_001492783.2 | | 8e-52 | |
| HMGIn60110 | CTACATATCAATGGAGCCTAAACTCACATTTTGCAAGTGGATAACTGAACACTGTCTTCCTCCCATGCCCTCCCTCACCAGG**[C/T]**AAAGAAAGCCCCAGGNTTGCTATGTGACTGAGCAAAGGGACAACCCCAGTGAAAGGCTGAGAACA | 3-hydroxy-3-methylglutaryl-coenzyme A reductase | NW_001492783.2 | | 2e-57 | |

^A^NCBI Reference Sequence ID ,

^B^E-value obtained from BLASTn against *Bos taurus* genome
